# A route to self-assemble suspended DNA nano-complexes

**DOI:** 10.1038/srep21995

**Published:** 2016-02-25

**Authors:** Yves Lansac, Jeril Degrouard, Madalena Renouard, Adriana C. Toma, Françoise Livolant, Eric Raspaud

**Affiliations:** 1 GREMAN, Université François Rabelais, CNRS UMR 7347, 37200 Tours, France; 2 Laboratoire de Physique des Solides, CNRS, Université Paris-Sud, Université Paris Saclay, 91405 Orsay cedex, France; 3 School of Materials Science and Engineering, Gwangju Institute of Science and Technology, Gwangju 61005, Korea

## Abstract

Highly charged polyelectrolytes can self-assemble in presence of condensing agents such as multivalent cations, amphiphilic molecules or proteins of opposite charge. Aside precipitation, the formation of soluble micro- and nano-particles has been reported in multiple systems. However a precise control of experimental conditions needed to achieve the desired structures has been so far hampered by the extreme sensitivity of the samples to formulation pathways. Herein we combine experiments and molecular modelling to investigate the detailed microscopic dynamics and the structure of self-assembled hexagonal bundles made of short dsDNA fragments complexed with small basic proteins. We suggest that inhomogeneous mixing conditions are required to form and stabilize charged self-assembled nano-aggregates in large excess of DNA. Our results should help re-interpreting puzzling behaviors reported for a large class of strongly charged polyelectrolyte systems.

Diverse suspended aggregates can be formed in solution by interaction of oppositely charged components. Let us mention for example hexagonal polymer-cationic surfactant complexes^1^,^2^, hollow icosahedron made of catanionic surfactants^3^, DNA-dendrimers or DNA-lipopolyamines complexes^4^,^5^,^6^. Such nano-aggregates have been often obtained with biological components and their structure has been explored (see for example^7^,^8^,^9^,^10^). Their defined size and charge are of utmost importance for many purposes in diverse fields of applications ranging from cosmetics or gene-therapy to paintings, waste-water treatment or paper industry^11^. Methods of preparation are critical to form these complexes, and may be difficult to reproduce because multiple parameters come into play such as mixing order, concentration of components before mixing, temperature, etc.^12^. Despite several theoretical^13^,^14^,^15^,^16^,^17^,^18^,^19^,^20^ as well as simulation studies^21^,^22^,^23^,^24^,^25^ focusing specifically on the formation of polyelectrolytes–multivalent ion complexes, the mechanisms involved remain mostly mysterious and need to be elucidated. We focus here on DNA because it is an extremely well controlled and widely investigated polymer with a high charge density that makes it an archetypal strong polyelectrolyte. Its condensation is directly relevant to genome packaging *in vivo* and to formulation of non-viral vectors for gene therapy. Protamines, responsible for sperm chromatin condensation, have been chosen for their precise amino acid composition and high charge density (21 over 30 amino acids are positively charged in salmon protamine). Soluble DNA-protamine complexes formed at low DNA and salt concentration as established in previous studies^26^,^27^,^28^ remain stable for months. We focus here on the mechanisms involved in their formation and stability by using a combination of multiple experimental and simulation approaches.

## Results

### A set of experimental analyses to study the nano-bundles

Previous light scattering studies^26^,^27^ showed that DNA and protamine molecules diluted in an aqueous solution of low ionic strength may form soluble complexes of dozens of nanometer size. This soluble state contrasts with the macroscopic phase separation observed at the isoneutrality point for which the charge ratio *R*_+/−_ (defined as the ratio of the protamine charges to the DNA phosphate charges) was found equal to 0.85, a value slightly lower than the nominal isoneutrality *R*_+/−_ = 1. We are interested here in studying the soluble DNA-protamine complexes formed in the two regions of solubility, namely in excess of DNA (*R*_+/−_ < 0.85) and in excess of protamines (*R*_+/−_ > 0.85) when a small drop of a concentrated protamine solution is directly added to the DNA solution. Previous measurements^26^,^29^ indicated that the complexes are negatively charged in excess of DNA and positively charged in excess of protamines. The charge ratio has been varied from 0.1 to 2 and we worked with DNA solutions diluted up to 0.01 g/l (30 μM Phosphate units) for TEM and electrophoresis experiments, and up to 0.03 g/l for light scattering studies. The mixture was stirred a few seconds and depending on the experiments, samples were analysed immediately or first concentrated by ultrafiltration before the analysis (see details in the Material and Methods section). The alternative mixing order (a drop of concentrated DNA added to a dilute protamine solution) was avoided for the reason that DNA chains in concentrated solution assemble into large precipitating aggregates as soon as they are in contact with protamines molecules even at low *R*_+/−_ values (see Fig. 4 in ref. 27). Moreover, handling concentrated solutions of DNA or oligonucleotides raises specific problems such as high viscosity and liquid crystalline states that must be avoided in formulation processes.

### Electrophoresis

**Figure 1:**
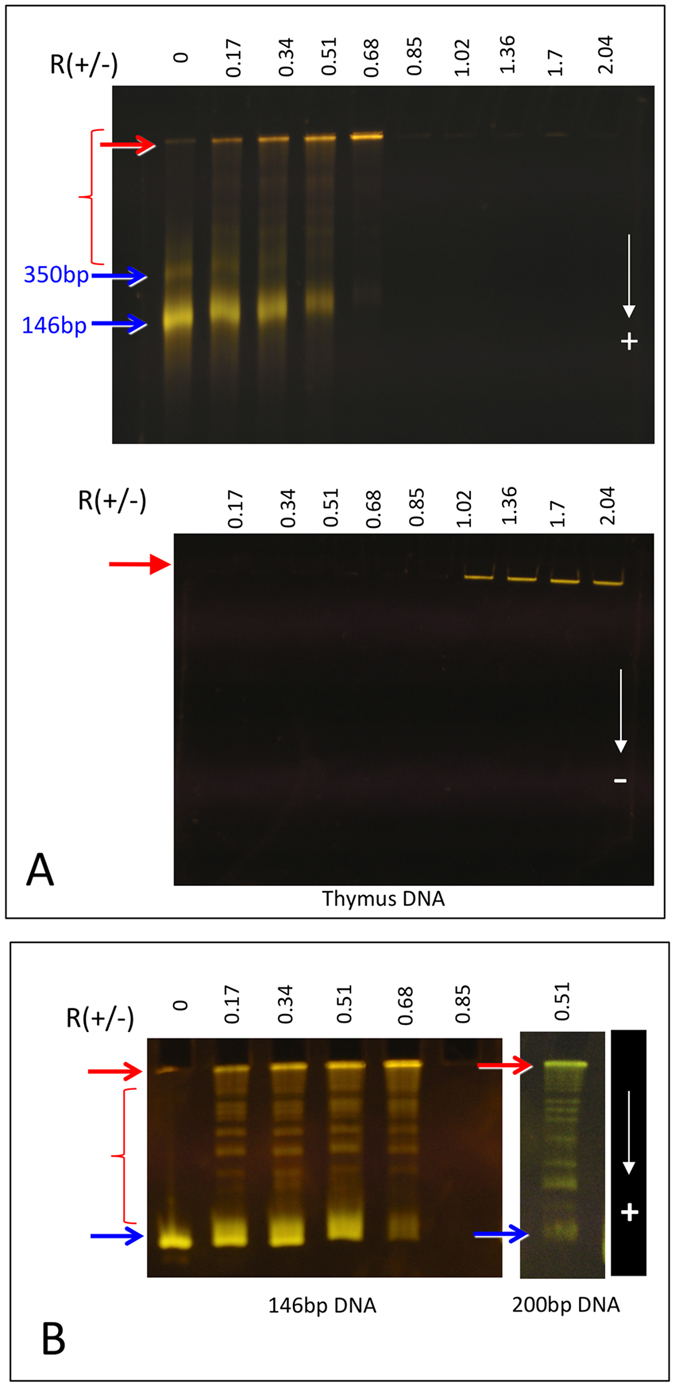
DNA-protamines complexes. **(A)** Electrophoretic patterns recorded for 0.1 < *R*_+/−_ < 2. The same series of samples prepared with nucleosomal DNA from calf thymus was migrated towards the positive (top) or towards the negative electrode (bottom). **(B)** Electrophoretic patterns recorded for *R*_+/−_ < 0.85 with perfectly monodisperse DNA fragments. Free DNA (blue arrows) and DNA-protamine complexes migrate towards the positive electrode as discrete bands (red marks) or remain stuck in the wells (red arrows).

Under an electric field, molecular species migrate in a polyacrylamide gel as a function of their charge and size. The mobility reflects the bare charge of DNA plus that of their counterions at its surface and its sign is expected to reverse on charge inversion. We used nucleosomal DNA fragments to prepare samples for a large range of *R*_+/−_ values (0.1 < *R*_+/−_ < 2). The samples were deposited in the wells and let to migrate either towards the positive or towards the negative electrode as indicated on Fig. 1A. Pure DNA (blue arrows, *R*_+/−_ = 0) as well as complexes formed from DNA/protamines mixture prepared at ratio *R*_+/−_ < 0.85 (red marks) migrate towards the positive electrode. A few larger complexes, also negatively charged, remain trapped in the wells (red arrows). Their amount increases when we get closer to *R*_+/−_ = 0.85. In excess of protamines (*R*_+/−_ > 0.85), all complexes are found in the wells only when migration is turned towards the negative electrode. They are positively charged and too large to migrate into the gel under our experimental conditions. Note that there is no isolated DNA fragments overcharged by protamines.

Because of the polydispersity of nucleosomal DNA and contamination with a minor amount of dinucleosomal DNA (350bp, blue arrows, Fig. 1A) it was difficult to analyze precisely the pattern of bands made by complexes migrating in the gel in excess of DNA. We thus prepared perfectly monodisperse DNA fragments (146 bp or 200 bp) (Fig. 1B). For *R*_+/−_ < 0.85, pure DNA that migrates as a single band (blue arrow) coexists with DNA-protamine complexes that are small enough to migrate into the gel (5–20 nm mesh size)^30^ towards the positive electrode. These complexes (red accolade) are visualized as discrete bands that draw the same pattern regardless of the charge ratio; only the relative intensities of the bands differ suggesting a different quantitative distribution of the same population of complexes (Fig. 1B). Most likely these bands reveal the presence of complexes made either by a single DNA fragment associated to an unknown number of protamines that weakly slow down their migration (decrease of their global charge and/or increase of their mass) or by a small number of DNA fragments glued by protamines. The electrophoretic pattern of negatively charged soluble complexes depends on the length and charge of the DNA fragments (compare patterns formed with 146 versus 200 bp DNA). Larger complexes remain stuck in the wells (red arrows) and their relative amount increases with the charge ratio up to *R*_+/−_ = 0.85. At this point, all DNA is precipitated and does not migrate in the gel anymore.

### Light scattering

**Figure 2:**
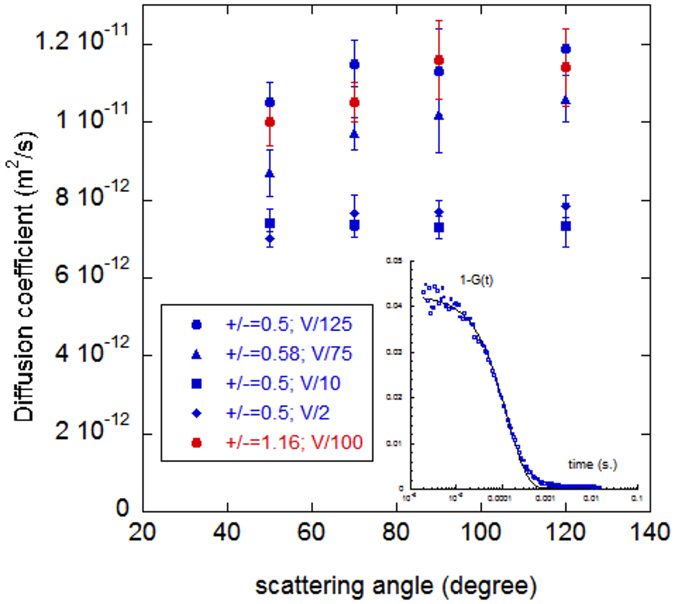
Bundle diffusion. Bundle diffusion coefficients measured by dynamic light scattering as a function of the scattering angle for samples prepared in excess of DNA (blue) and in excess of protamines (red). The volume of the added protamine droplet was varied from *V/2* to *V/125*, with *V* the final volume of the sample. Inset: typical auto-correlation function.

Dynamic light scattering experiments were performed to confirm the existence of these suspended objects. Auto-correlation functions of the scattered intensity were measured at different angles and present only single exponentials that we attribute to the diffusion of the largest complexes because of their predominant contribution to the detected signal. The diffusion coefficients of the complexes, extracted from the decay times, are found nearly constant in the explored angular range (Fig. 2). Values are in the same range for samples prepared in excess of DNA and in excess of protamines. From the averaged value *D* of the diffusion coefficients, a hydrodynamic radius *R*_*H*_ of 30 ± 5 nm is obtained if we consider the hypothesis of a spherical object (*D* proportional to *1/R*_*H*_). If we consider a cylindrical bundle structure of length *L* equal to the DNA contour length (*D* proportional to (*ln (d*_*f*_*/L))/L*), its diameter *d*_*f*_ would be 20 ± 5 nm. Taking into account the interhelix distance measured by cryoTEM (see the next paragraph), we may estimate that the average number of DNA fragments in a bundle would be of the order of 50, meaning that less than ten DNA fragments are aligned side by side along the bundle diameter. No significant change was observed in measurements repeated two weeks later. These objects with a given size polydispersity would correspond to the complexes trapped in the wells of the acrylamide gels. Interestingly, we noticed that the size of the complexes changes when the volume of the added protamine droplet is varied while keeping the final ratio constant, with *R*_+/−_ = 0.5 (excess of DNA) (Fig. 2).

### Cryo-TEM

**Figure 3:**
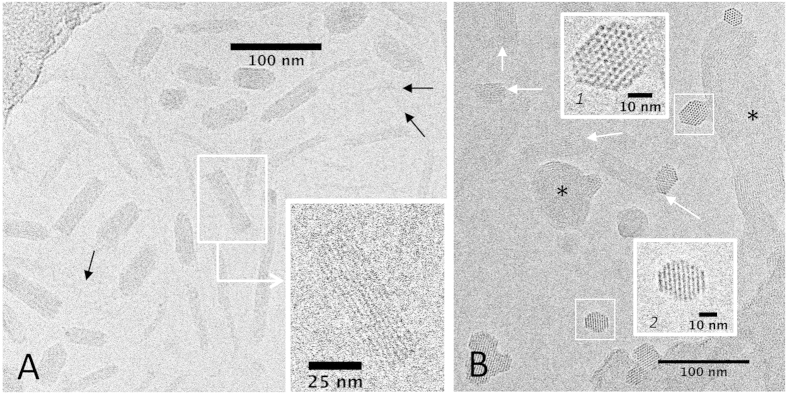
DNA-protamines complexes observed by cryoTEM. **(A)** Complexes formed in a monodisperse 200 bp DNA fragment solution prepared at *R*_+/−_ = 0.51, and left 8 days to stabilize after concentration by filtration. Bundles of multiple diameters are mostly seen here in side or slightly oblique views. Black arrows point to the smallest ones and/or to isolated DNA fragments. (**B)** Complexes formed with nucleosomal DNA at *R*_+/−_ = 1.36 and immediately frozen after concentration. Bundles are seen in side view (white arrows), or in top view (insert 1 for example) or in slightly oblique view (insert 2) and coexist with larger aggregates (*). Images are under-focused by 2–3 μm in (**A**) or 800 nm (in **B** and inserts) to visualize structural details.

To visualize soluble complexes, we used cryoTEM, a method that provides a direct observation of the samples in their native state. Figure 3A shows complexes prepared at a charge ratio *R*_+/−_ = 0.51 (excess of DNA). The length of the bundles is close to the length of the DNA fragments (200 bp, 68 nm), with no significant longitudinal shift of aligned fragments. Arrows in Fig. 3A point to thin filaments that may be single naked DNA fragments, single DNA decorated with protamines or bundles made of 2 or a very few number of chains. These complexes are remarkably stable over time: no aggregation was detected after one month in samples concentrated up to 60x. We assume that the largest bundles seen on Fig. 3A are the complexes stuck in the wells in Fig. 1A,B.

Figure 3B shows complexes formed in excess of protamines (*R*_+/−_ = 1.36). Bundles present multiple orientations. Some bundles are parallel or slightly oblique to the film (white arrows) with striations visible only under favorable orientation. They look similar to the bundles formed in excess of DNA. Top views reveal their hexagonal structure and the facetted sections of the bundles (detail in insert 1 for example). A slight deviation from a perfect top view turns the hexagonal lattice into striated patterns of period *d* = *a*_*H*_ *√3/2*, with *a*_*H*_ the interhelix distance (insert 2). These bundles coexist with larger aggregates (indicated by asterisks on Fig. 3B) showing locally the same striated pattern with the same period. They are most likely formed by aggregation of isolated bundles such as those seen closeby.

### Bundle structure revealed by Cryo-TEM

**Figure 4:**
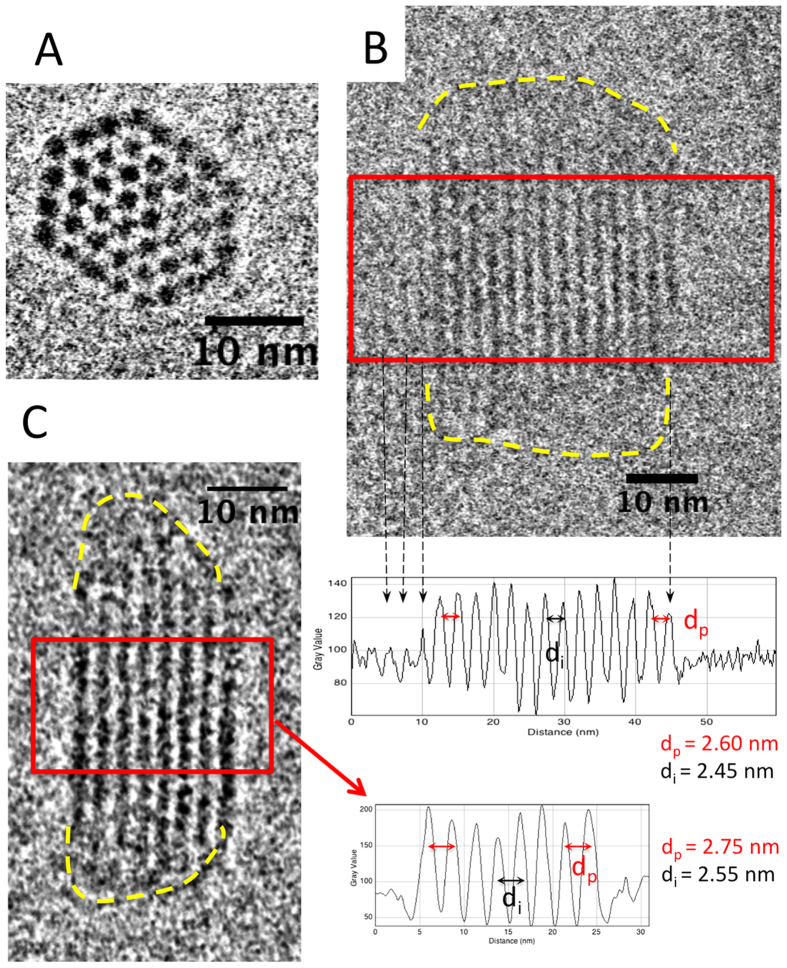
Structure of the hexagonal DNA-protamine bundles. Bundles formed by 146 bp DNA fragments for *R*_+/−_ = 1.36 **(A,C)** and by 200 bp DNA for ratio *R*_+/−_ = 0.51 **(B).** Intensity profiles of regions framed in red are used to measure the periods *d*_*i*_ inside the bundles and *d*_*p*_ at their boundary. Dotted arrows on the micrographs and on the profiles **(B)** point to peripheral layers that are often of lower contrast, less precisely defined, and more difficult to follow over the entire length of the bundle. Extremities of the bundles, devoid of striations are underlined by dotted lines. CryoTEM observations are performed at low underfocus (800 nm).

The structural details of the bundles formed in excess of DNA (*R*_+/−_ = 0.51) and in excess of protamines (*R*_+/−_ = 1.36) have been investigated at low defocus to achieve a high cryoTEM resolution (Fig. 4). Top views reveal the facetted compact shape of the bundles and the hexagonal DNA lattice (Fig. 4A) while side and oblique views often present a network of parallel lines along the axis of the bundle (Figs 3A, insert and 4B,C). These stripes are 2D projections of the reticular planes of the hexagonal lattice separated by a distance *d* = *a*_*H*_*√3/2* as described earlier^31^,^32^. Precise measurements of the periodicities were carried out from intensity profiles recorded on selected domains on a series of bundles (examples framed in Fig. 4B,C, with their corresponding profiles). Peripheral DNA layers present noticeable particularities: i) they are kept at a distance *d*_*p*_ larger than the distance *d*_*i*_ between layers in the core of the bundle. For *R*_+/−_ = 0.51 (excess of DNA), the mean interhelix distance is *a*_*H*_ = 2.85 ± 0.10 nm, with *a*_*H*_ = 2.95 ± 0.10 nm at the periphery compared to *a*_*H*_ = 2.78 ± 0.10 nm in the core of the bundle. For *R*_+/−_ = 1.36 (excess of protamines), the effect is more pronounced with *a*_*H*_ = 2.50 ± 0.10 nm in the core and *a*_*H*_ = 3.10 ± 0.10 nm at the periphery (mean *a*_*H*_ = 2.90 ± 0.10 nm) ii) peripheral layers may also be less contrasted, and less extended longitudinally which produces a lower density on the profiles. Moreover the lack of striations observed quite often at the extremities of the bundles (limits underlined by dotted lines in Fig. 4B,C) may result either from an unfavorable orientation of the hexagonal lattice or from a less ordered structure. Structural details of the bundles extremities are thus missing.

### Molecular simulations

**Figure 5:**
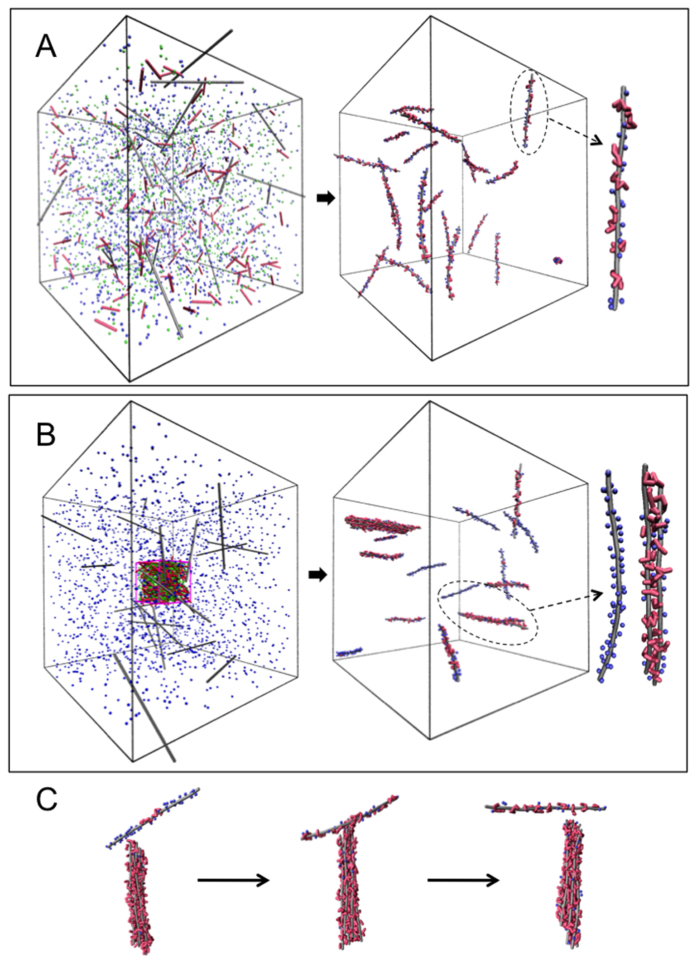
Bundle formation and dynamics at low protamine concentration (*R*_+/−_ = 0.5). (**A)** From initial *homogeneous* conditions, the system evolves towards a state with mostly isolated DNA (gray rods) roughly decorated evenly by protamines (red chains) and counterions (blue balls). (**B**) From initial *inhomogeneous* mixing conditions consisting of a small droplet of protamines with their counterions (green balls) injected in the solution, the system evolves towards a state with a coexistence of negatively charged bundles, isolated decorated DNA and isolated naked DNA. (Langevin dynamics with *N*_*DNA*_ = 20 and *N*_*PRO*_ = 143 in a cubic container of side *L* = 48 nm and a droplet of volume *v* = *V*/100). **(C)** Transient binding of a poorly decorated DNA resulting in a significant increase of the (negative) charge of the bundle formed in (**B**) through protamine transfer.

The microscopic mechanisms at the origin of the bundle formation are investigated through molecular simulations using a coarse-grained model of the DNA, the protamines and their counterions. Species interact solely through long-range electrostatic and steric interactions and water is treated as a continuum dielectric medium (see Methods). Although the full range of *R*_+/−_ charge ratio has been explored, a special emphasis is given to the unexpected and intriguing stabilization of negatively charged bundles observed at low charge ratio. The dynamic evolution of a dilute DNA solution (~30 mM) prepared at *R*_+/−_ = 0.5 under different initial conditions is investigated. The starting state of the first simulation corresponds to a *homogeneous mixing* situation where both DNA fragments and protamines are randomly distributed in a volume *V* (Fig. 5A). A large fraction of monovalent counterions initially adsorbed on each DNA are very quickly (~3 ns in the simulation time scale) replaced by protamines. Using a similar bead-spring model, Ou and Muthukumar^33^ have explicitely demonstrated that the complexation between two oppositely-charged strong polyelectrolytes was indeed driven by the entropy gain coming from the release of a large amount of counterions (see also the theoretical work from Record *et al.*^34^). This exchange is driven mostly by the positional entropy gain of the monovalent counterions outweighing the positional and conformational entropy loss of the protamines. All protamines are adsorbed onto DNA but no DNA self-assembly is observed: the final state is constituted by isolated DNA decorated by the same average number of protamines.

The starting state of the second simulation mimics the *inhomogeneous mixing* resulting from the injection of a protamine droplet (see Methods) by confining all the protamines and their counterions within a small sub-volume *v* = *V*/100 inside the DNA solution. DNA fragments within and around the sub-volume experience a micro-environment characterized by a large excess of protamines, i.e *r*_+/−_ = (*V/v*)** **×** ***R*_+/−_ = 50. Species diffusions are slow enough to allow a large amount of protamines to adsorb onto the DNA located within the micro-environment leading to the formation of bundles (Fig. 5B and Supplementary movie S1). Protamine adsorption and DNA self-assembly appear to be largely concomitant (see Supplementary movie S1). The DNA self-assembly occurs in ~3–4** **ns while the total adsorption of protamines onto DNA is completed in less than 1** **ns. DNA fragments start assembling into loose aggregates evolving rapidly towards well-ordered bundles with DNA aligned side-by-side through several molecular exchanges and rearrangements (Supplementary movie S1). Due to the local excess of protamines at the origin of their formation, loose aggregates are initially positively-charged but the reorganisation process is accompanied by an inversion of the bundle charge (Supplementary Fig. S1). We observed that the magnitude of the charge is further increased through a transient bridging mechanism: under thermal fluctuations, a naked or poorly decorated DNA fragment may approach and join the bundle from one of its ends and with a relative perpendicular orientation in order to lower the electrostatic repulsive barrier (Fig. 5C, Supplementary Fig. S1 and movie S2). Some protamines are transferred from the bundle surface to the naked DNA fragment resulting in an increase of the bundle negative charge and consequently to the unbinding of the bundle and the fragment due to repulsion. Eventually negatively charged bundles coexist with isolated DNA decorated by a variable number of protamines and with isolated naked DNA that were initially far from the micro-environment (Fig. 5B).

The same dilute DNA solution prepared at a smaller excess of DNA (e.g. *R*_+/−_ = 0.8) under homogeneous mixing conditions forms soluble (negatively-charged) bundles coexisting with isolated DNA fragments decorated by the same amount of protamines. At the isoelectric point (*R*_+/−_ = 1) and under the same conditions, all the DNA chains assemble into a single neutral aggregate that might correspond to the dense phase occurring in the macroscopic phase separation. In a surplus of protamines (*R*_+/−_ > 1), the dilute DNA solution prepared either under homogeneous or inhomogeneous mixing conditions forms positively-charged bundles coexisting with free-protamines. These bundles tend to aggregate over time by approaching each other and merging near their tips with a relative perpendicular orientation in order to minimize the electrostatic repulsions.

### Microscopic organization in the bundles

**Figure 6:**
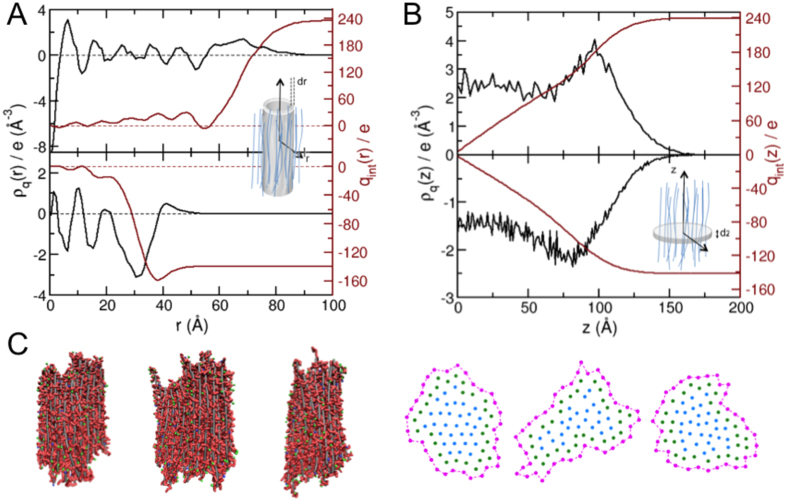
Simulated bundle structure. **(A)** Radial charge density *r*_*q*_ *(r)* (black line, scaled up by 2 × 10^4^) located in a cylindrical volume of the bundle with a radius *r* and a thickness *dr* (see shaded volume on the scheme) and integrated radial charge 
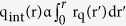
 (red line) for a bundle in excess of protamines (*N*_*DNA*_ = 91, *R*_+/−_ = 2.0) (top) and in excess of DNA (*N*_*DNA*_ = 27, *R*_+/−_ = 0.8) (bottom). (**B)** Longitudinal charge density *r*_*q*_(z) (black line, scaled up by 2 × 10^5^) located in a disk-like volume of thickness *dz* and arbitrary radius located at a position *z* within the bundle (see shaded volume on the scheme) and integrated longitudinal charge 
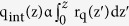
 (red line) for the bundle in excess of protamines (top) and in excess of DNA (bottom). The bundle center of mass is located at *z* = 0. (**C)** Snapshots showing the longitudinal and the lateral fluctuations (second peripheral DNA “layer” colored in green and core in blue) in the pre-formed bundle in excess of protamines for *R*_+/−_ = 2.0.

Pre-formed large hexagonal bundles equilibrated by molecular dynamics for charge ratio close to (*R*_+/−_ = 0.8) or above (*R*_+/−_ = 2) the isoelectric point have been analyzed in detail. They exhibit an alternating positive and negative density of charge. The bundle core is therefore mostly neutral to insure cohesion (Fig. 6A) while its surface provides the main contribution to the total charge. The charge is positive for the bundle formed at *R*_+/−_ = 2 (Fig. 6A top) due to an excess of protamines on its surface while it is negative for the bundle formed at *R*_+/−_ = 0.8 due to a protamine deficit (Fig. 6A bottom, Supplementary Fig. S2). Along the bundle main axis the density of charge is roughly constant in the central part of the bundle and increases at the tips (Fig. 6B). Therefore, the bundles stabilized above the isoelectric point (*R*_+/−_ > 1) exhibit an excess of protamines at their tips while bundles stabilized below the isoelectric point (*R*_+/−_ < 1) exhibit a protamine deficit. In addition, a significant amount of protamine (resp. DNA) counterions are adsorbed on the charged surface of the bundles stabilized at *R*_+/−_ > 1 (resp. *R*_+/−_ < 1) (Supplementary Fig. S2). Bundles present a fluid-like structure with significant lateral and longitudinal fluctuations (Fig. 6C and Supplementary movie S3). Interestingly, the mean distance between nearest-neighbors as well as the sliding of DNA along each other are larger for the fragments located at the periphery of the bundle (Fig. 6C and Supplementary Fig. S3). Both effects are favored at the periphery compared to the core because the loss of electrostatic energy is minimized there due to the reduced number of neighbors.

## Discussion

### Kinetically trapped-states vs. equilibrium states

**Figure 7:**
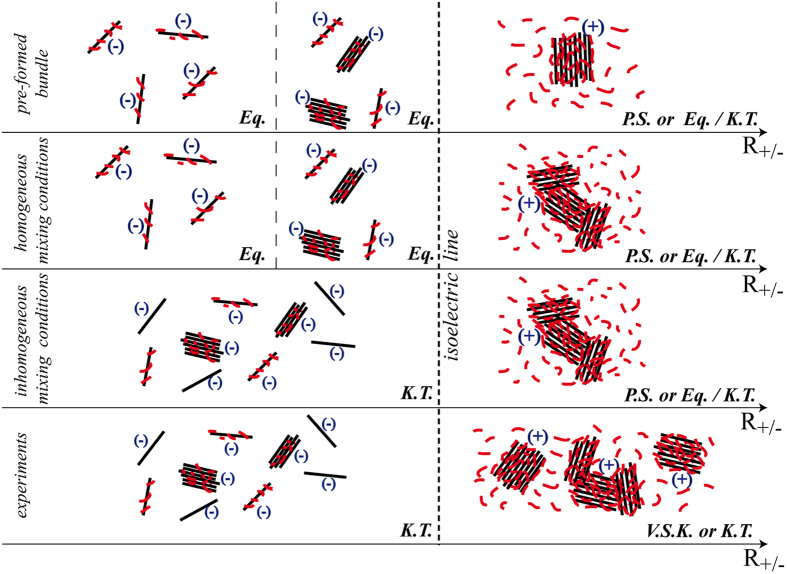
Schematic views of possible resulting states deduced from experiments and simulations. Depending on the initial conditions, final states may be constituted of bundles at equilibrium (Eq.), evolving very slowly (Very Slow Kinetics, V. S. K.) or kinetically trapped (K.T.). Simulations do not permit to rule out the possibility of a complete phase separation (P.S.) in excess of protamines. DNA are represented by black lines, protamines by red lines and the charge carried by an isolated DNA or a bundle is indicated in parentheses.

Figure 7 summarize the informations obtained by the combined experimental and modeling approach about the microscopic behavior of the system at different charge ratio. The formation of the bundles is the result of a sensitive interplay between the enthalpy gain and the entropy loss (coming in large part from DNA) upon self-assembly. The attempt to minimize the loss of entropy is clearly demonstrated by the dynamical nature of the bundles presenting a fluid-like structure with significant lateral and longitudinal fluctuations (Fig. 6C and Supplementary movie S3). The enthalpy contribution is related to the competition between short-range attraction vs. long-range repulsion. In the various proposed models^35^,^36^,^37^,^38^,^15^,^39^ the short-range attraction is associated in a way or another to the correlations in the fluctuations of the condensing agents adsorbed onto the polyelectrolytes.

Experimental observations as well as theoretical approaches^40^,^41^,^15^,^42^,^43^,^44^,^45^,^46^,^47^ estimate that the fraction of charge neutralization (*f*) must be at least larger than 50% with values most commonly comprised between 89% and 100% to induce attraction. In excess of protamines (*R*_+/−_ > 1) simulations under homogeneous as well as inhomogeneous mixing conditions show that positively-charged bundles exhibit a slow dynamics leading to aggregation over time. In addition, a pre-formed and equilibrated bundle (see Methods) at the same charge ratio remains stable. However, limitations in simulation sizes prevent to determine whether the aggregated state corresponds to a macroscopic phase separation or to a state (either equilibrium or kinetically-trapped) constituted by larger (than simulated) positively-charged bundles in solution. Experiments confirm that bundles are slowly evolving over time (several weeks) towards larger aggregates. Moreover the fact that isolated bundles observed in light scattering experiments turn partially aggregated after concentration of the sample (cryoTEM) suggests that bundles constitute a kinetically-trapped state. In excess of DNA, but close enough to the isoelectric point (*R*_+/−_ > 0.5, e.g *R*_+/−_ = 0.8) bundles are formed under homogeneous or inhomogeneous mixing conditions. A similar final state is obtained starting from a pre-assembled bundle suggesting that small negatively-charged bundles constitute an equilibrium state.

At low *R*_+/−_ ratio (*R*_+/−_ ≤ 0.5), simulations under homogeneous mixing conditions show clearly that the resulting fraction of charge neutralization (*f* ~ *R*_+/−_) is not enough to induce self-assembly. Therefore, the final state corresponds to negatively-charged isolated decorated DNA repelling each other (Fig. 7). Additional simulation performed at *R*_+/−_ = 0.5 starting from a pre-formed and equilibrated single bundle results in the same final state through the sequential and complete bundle disassembly (Supplementary Fig. S4). The disassembly of the pre-formed bundle is the result of a delicate balance: the gain in entropy (orientations, positions and conformations of DNA) is here larger than the loss in enthalpy (electrostatic energy) (right axis on Fig. S4). The fact that the same state is reached from significantly different initial conditions suggests that isolated DNA decorated with protamines are in equilibrium at low charge ratio. We therefore hypothesize that the nano-bundles observed experimentally at low protamine concentrations (*R*_+/−_ < 0.5) constitute a kinetically trapped state resulting from the initial spatial inhomogeneity of the species. The arguments supporting this hypothesis are the following:

1) Electrophoresis experiments show that bundles coexist with a large amount of naked DNA fragments surrounded by their monovalent counterions. Inhomogeneous mixing conditions and strong binding of protamines to DNA^47^ lead to a region rich in protamines (bundles) and a region depleted of protamines.

2) Light scattering experiments show that the bundle size depends of the size of the protamine droplet injected in the solution.

3) Bundles are still observed at very low protamine concentrations (*R*_+/−_ ~ 0.1) and they remain stable for weeks. Simulations performed under homogeneous mixing condition indicate clearly that a critical number of protamines per chain is needed to induce bundle formation. Inhomogeneous mixing conditions allow to *locally* satisfy this requirement.

4) Bundles formed at dilute DNA concentration remain stable through concentration by water and ions filtration while a mixture initially prepared at higher DNA concentration lead to a macroscopic phase separation.

### Size-limitation of the bundles

Many systems, such as colloids^48^, are controlled by the competition between short-range attraction and much longer-range repulsion which lead to finite-size aggregates in equilibrium. However the assumption that interactions are pairwise additive to explain such behavior does not hold in strongly charged rodlike polyelectrolytes at low salt concentration^49^,^50^ because the condensing agents within a growing bundle are constantly redistributing themselves, leading instead to a macroscopic phase separation if enough condensing agents are present. Another mechanism is therefore needed to explain finite-size bundles formed by DNA fragments. The short-range attraction mediated by adsorbed condensing agents is only effective when polyelectrolytes are nearly parallel to each other in order that their respective condensing agents can be correlated. If polyelectrolytes are significantly tilted away with respect to each other they will instead repel resulting in the existence of a free-energy barrier as a function of the angle. Such a barrier exists also between an already formed bundle and a single polyelectrolyte and it has been predicted by Ha and Liu^13^,^14^ that its height increases linearly with the size of the bundle: a polyelectrolyte arriving with a significant angle with respect to the bundle long axis will be repelled by the chains belonging to the facing surface of the bundle. As the bundle grows larger and larger a strong repulsion will prevent polyelectrolyte to approach it in the right orientation to be attracted. The very slow evolution of the bundles reported in experiments is consistent with the existence of a high energy barrier requiring long time to be crossed. Therefore strong electrostatic repulsion stabilize finite-size negatively-charged bundles coexisting with isolated DNA fragments formed in excess of DNA (*R*_+/−_ = 0.5 and *R*_+/−_ = 0.8) under inhomogeneous mixing conditions.

Positively-charged bundles formed in excess of protamines either under inhomogeneous or homogeneous mixing conditions tend to merge with relative perpendicular orientation. Such aggregation is therefore probably not driven by short-range attractions arising from correlations. Instead, the resulting gain in entropy resulting from the observed release of a fraction of the protamines adsorbed onto the surfaces of the merging bundles might outweigh the repulsive interaction to allow aggregation. Other mechanisms such as depletion interaction induced by the excess of charged protamines remaining in solution or bridging interactions between bundles mediated by poorly decorated DNA (especially in the case of inhomogeneous mixing conditions) might also play a role. Finite-size bundles or micelles have also been predicted at thermal equilibrium as a result of geometrical constraints or interactions arising for example from excluded volume or correlations^16^,^51^,^52^. For instance Huang & Olvera de la Cruz^18^ found that finite-size aggregates are stabilized by the chain entropy which would be otherwise strongly reduced due to stretching upon an increase in the aggregate size. The aggregates resulting from this micellization process are predicted to exist within a very limited range of charge ratio *R*_+/−_ in contrast to our experimental situation where bundles are observed within an extended range. These aggregates could however correspond to the bundles formed in simulation performed under homogeneous mixing condition either in excess of DNA near the isoelectric point (0.5 < *R*_+/−_ < 1) or in excess of protamines (*R*_+/−_ > 1). Coarse-grained simulations performed by Sayar and Holm^21^,^22^ may also suggest that finite-size bundles can be stabilized in a narrow range of Bjerrum lengths by tuning the solvent dielectric constant. As pointed out by Muthukumar and collaborators^53^, the omnipresent primary aggregates observed experimentally in the process of classical nucleation of a phase leads to a new mechanism of phase separation in polyelectrolyte systems.

Finally, we note that the amplitude of the fluctuations observed at the peripheries and tips (Fig. 6C, Supplementary Fig. S3 and Movie S3) might also play an important role in controlling the bundle size. This effect is different from the classical role played by a surface energy^54^ that tends to reduce the surface area, to limit the size of a nucleus, to favor a flat surface without roughness and to decrease the regular spacing. Here the fluctuations increase the roughness and the local spacing between peripheral DNA.

We have shown that inhomogeneous mixing conditions constitute a robust route to self-assemble polyelectrolytes. Interestingly, a somehow similar inhomogeneous mixing process may also lead to frozen multilayer polyelectrolyte films^55^, revealing a strong analogy with the kinetically trapped DNA bundles obtained in this study at very low charge ratio *R*_+/−_. These bundles may inspire the conception of new engineered nano-systems in the future. Pre-designed nanostructures could be built by using either nanofluidic devices or localized injections of condensing agents through computer-controlled glass nanocapillars in a diluted polyelectrolyte solution. In particular, condensing agents having electronic properties (such as functionalized gold particles and conjugated oligo-electrolytes) or optical properties (such as CdSe luminescent nanorods) would allow to build nanostructures useful in the emerging fields of nanoelectronics and nanophotonics.

## Methods

### Materials

Nucleosomal DNA (146 bp ± 5 bp plus a fraction of dinucleosomal DNA (350 bp)) was prepared by enzymatic digestion of calf thymus chromatin, as described previously^56^,^57^. Monodisperse 146bp and 200bp DNA fragments correspond respectively to the sea urchin 5S rRNA gene and to the 601.5 sequence. Stock solutions of DNA and salmon protamine (Sigma, grade X) were dialyzed first in 2 M NaCl to replace all counterions by sodium. DNA and protamine concentrations were determined by UV absorption (*A*_*260*_ = 1 when *C*_*DNA*_ = 0.15 mM and *A*_*230*_ = 1 for *C*_*protamine*_ = 0.47 g/l). Complexes were formed by addition of small volumes of protamines (10 mM Tris, pH 7.6) to DNA (2 or 10 mM Tris, pH 7.6) at a final DNA concentration of 0.01 g/l for electrophoresis and cryoTEM experiments and 0.03 g/l for light scattering. For light scattering experiments, the size (and concentration) of the protamine drop was varied to reach a given *R*_+/−_ while keeping constant the final DNA concentration. The ratio *R*_+/−_ corresponds to the concentration of (+) charges carried by protamines over the concentration of (−) charges carried by DNA (2 charges/bp), considering that DNA and protamines are fully charged under our buffer conditions.

### Characterization

Electrophoreses of the soluble fraction were performed at 150 V for 40–60 min in 5% polyacrylamide gels in 18 mM Tris Borate buffer, pH 8.2 and gels stained by SYBRGreen.

For light scattering measurements, samples were analyzed in a home-made set-up^58^. Great care was taken to remove dust particles by filtering the buffers. The detected signals were at least three times the buffer signal and remained constant.

**Figure 8:**
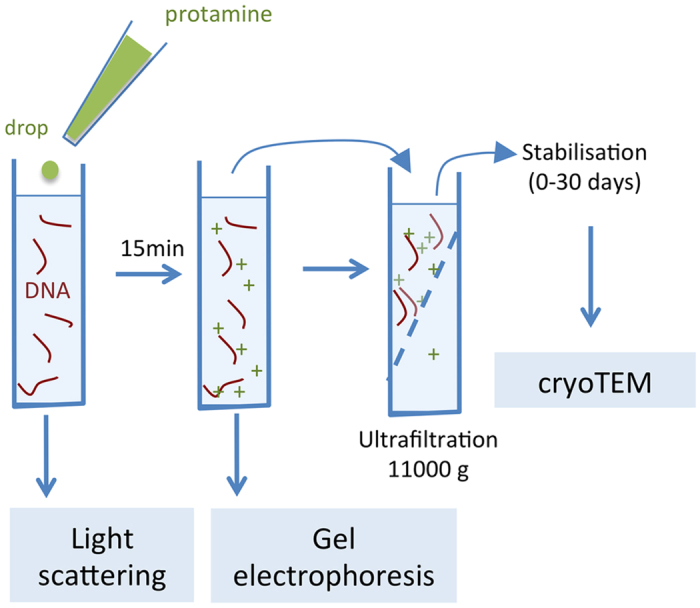
Experimental protocol. Schematic drawing of the protocol followed to prepare the samples used in the three experimental methods of characterization.

For cryoTEM observations, samples prepared at two ratios *R*_+/−_ = 0.51 and *R*_+/−_ = 1.36 have been concentrated up to 60x by ultrafiltration (Millipore, MWCO 10KD, 4-11000 g) to reach DNA concentrations needed for EM observations (0.6 to 1.2 g/l), let to stabilize up to one month and vitrified under controlled conditions of temperature and hygrometry. Images were recorded in a JEOL 2011 cryoTEM at 50000X magnification. Figure 8 summarizes the methodological details.

### Molecular simulations

Polyelectrolytes are modeled with a coarse-grained bead-spring model. Each bead of a DNA fragment carries a negative unit charge while each bead of a protamine carries a positive unit charge. A harmonic bond potential insures chain connectivity. Equilibrium bond length is set to 1.7 Å for DNA and to 5.6 Å for protamine in order to reproduce the charge densities. Intrinsic stiffness of DNA is enforced through a harmonic bond angle potential with an equilibrium bond angle set to 180^o^ while protamine is modeled as an intrinsic fully flexible chain. Counterions are explicitly treated as single beads carrying either a positive or negative unit charge. Non-bonded beads interact through long-range electrostatic interactions. Excluded-volume effects between beads are reproduced by a short-range repulsive potential (Weeks-Chandler-Andersen). The diameter of any bead is set to 4 Å. Due to computational limitation, the polyelectrolyte lengths are reduced by one third resulting in chains made of 100 and 7 beads for DNA and protamines respectively. Water is treated as a uniform dielectric background and no salt is added. The dynamics of the system is explored at constant room temperature and in very dilute conditions (20 DNA fragments in a 480 Å side cube) using Langevin dynamics in order to reproduce the translational diffusion coefficients of DNA and protamine. Note however that the system size corresponds to a DNA solution x1000 times more concentrated than the experimental concentrations. Additional simulations are performed starting from preformed hexagonal bundles for different charge ratios *R*_*+/−*_ = 0.5, 0.8, and 2.0. The simulations proceed in two stages: in the first stage (equilibration) the DNA are kept fixed and completely rigid on the hexagonal lattice while the protamines and the counterions are allowed to distribute themselves inside the bundle; in the second stage (production) the system evolves freely. All the simulations are performed using the molecular package LAMMPS^59^.

## Additional Information

**How to cite this article**: Lansac, Y. *et al.* A route to self-assemble suspended DNA nano-complexes. *Sci. Rep.* **6**, 21995; doi: 10.1038/srep21995 (2016).

## Supplementary Material

Supplementary Movie S1

Supplementary Movie S2

Supplementary Movie S3

Supplementary Information
