# Supplementary material for: A route to self-assemble suspended DNA nano-complexes
Source: Sci Rep. 2016 Feb 25;6:21995. doi: 10.1038/srep21995 (PMC4766487; doi:10.1038/srep21995)
Supplement: Supplementary Information [file srep21995-s4.pdf]

Supplementary Information

A route to self-assemble suspended DNA nano-complexes

Yves Lansac[1,2,3] , Jeril Degrouard[2], Madalena Renouard[2, †], Adriana C. Toma[2], Françoise Livolant[2] & Eric Raspaud[2]

[1] GREMAN, Université François Rabelais, CNRS UMR 7347, 37200 Tours, France.
[2] Laboratoire de Physique des Solides, Université Paris Saclay, CNRS UMR 8502, 91405 Orsay cedex, France.
[3] School of Materials Science and Engineering, Gwangju Institute of Science and Technology, Gwangju 500-712, Korea.

† present address: Institute for Integrative Biology of the Cell, Université Paris Saclay, CNRS UMR 9198, 91405 Orsay cedex, France.

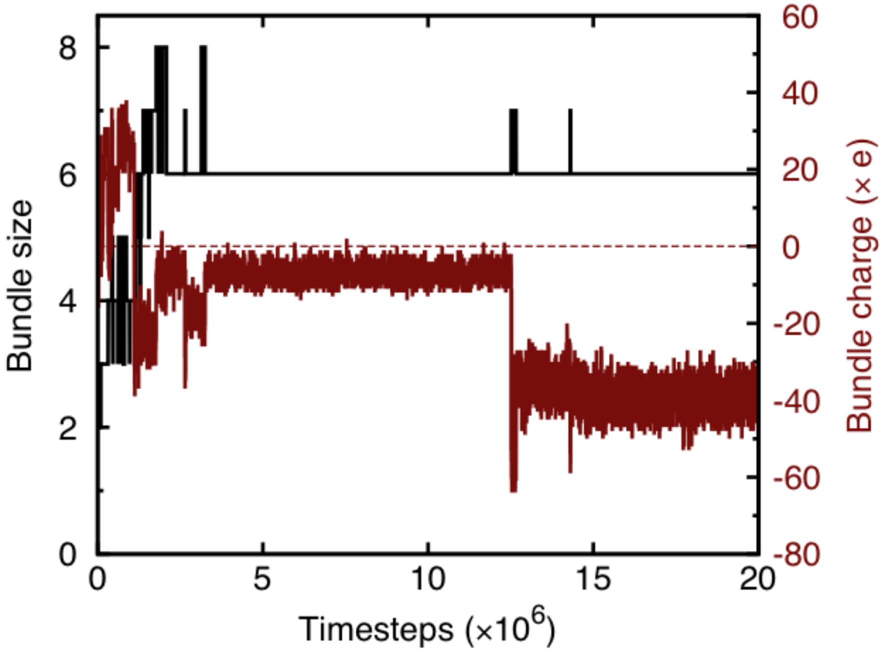

Figure S1. Time evolution of the size (number of DNA chains, black) and charge (red) of the largest bundle formed from inhomogeneous mixing condition for $R_{+/-}$ = 0.5**.**

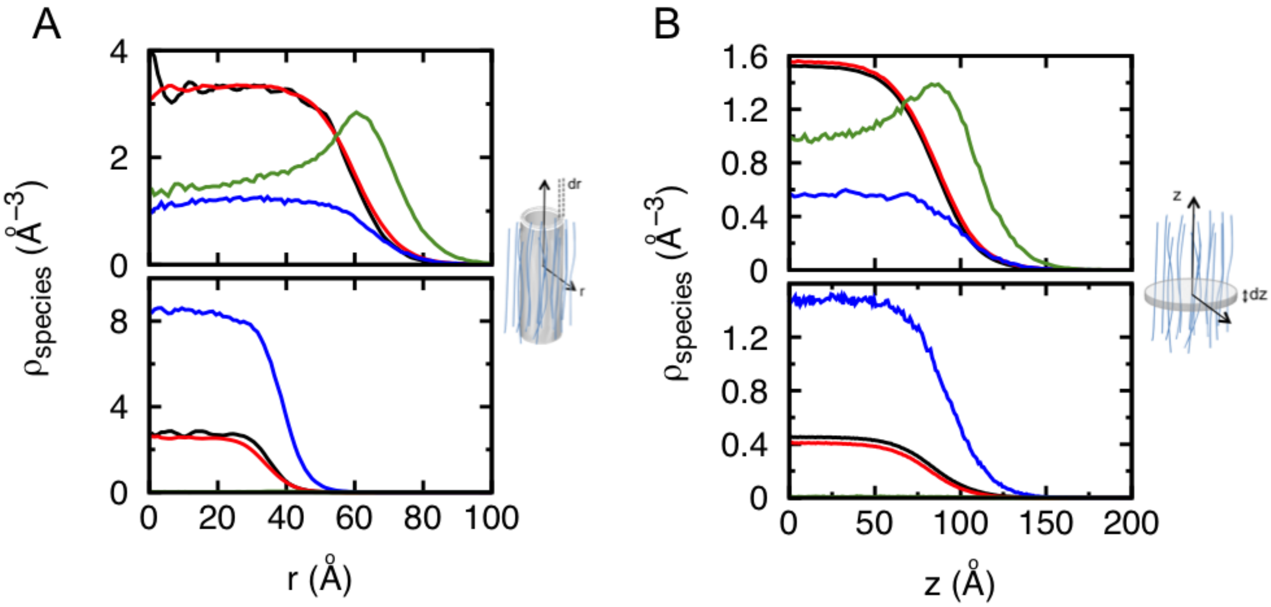

Figure S2. A. Radial number density $\rho_{species}$ of each species located in a cylindrical volume of the bundle with a radius r and a thickness *dr* (shaded volume on the scheme) for a bundle in excess of protamines (N_{DNA} = 91, $R_{+/-}$ = 2.0) (top) and in excess of DNA (N_{DNA} = 27, $R_{+/-}$ = 0.8) (bottom). Densities are represented in black for DNA, red for protamine, blue for DNA counterions and green for protamine counterions. **B.** Longitudinal number density $\rho_{species}$ located in a disk-like volume of thickness *dz* and arbitrary radius located at a position *z* within the bundle (shaded volume on the scheme). The bundle center of mass is located at z = 0. Densities are represented in black for DNA (scaled up by 2×10^5), red for protamine, blue for DNA counterions (×50) and green for protamine counterions (×50).

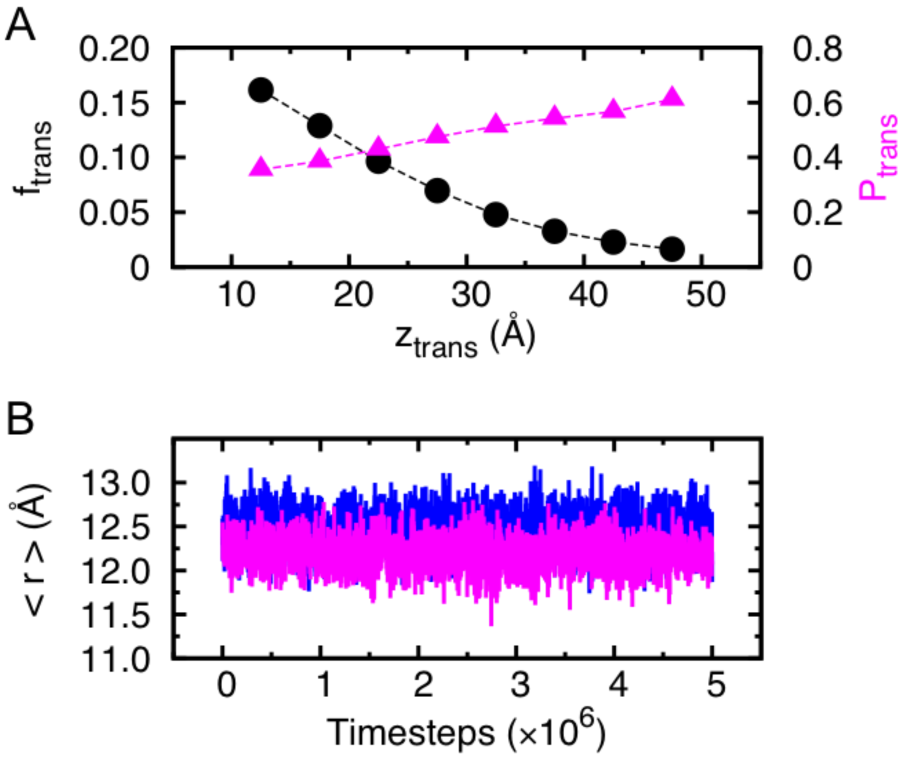

Figure S3. **A**. Longitudinal fluctuations in the pre-formed bundle in excess of protamines. Black circles indicate the fraction f_{trans} of DNA molecules translated longitudinally by $z_{trans} \pm \Delta z$ ($\Delta z = 2.5$ nm) with respect to the bundle center of mass. P_{trans} is the probability that the translation involves DNA located on the periphery of the bundle (magenta triangles). **B.** Time evolution of the average distance between two DNA located in the bundle core (blue, on average ~12.45 ± 0.01) *versus* the distance between a peripheral DNA and its nearest neighbors in the bundle core (magenta, on average ~ 12.18 ± 0.01).

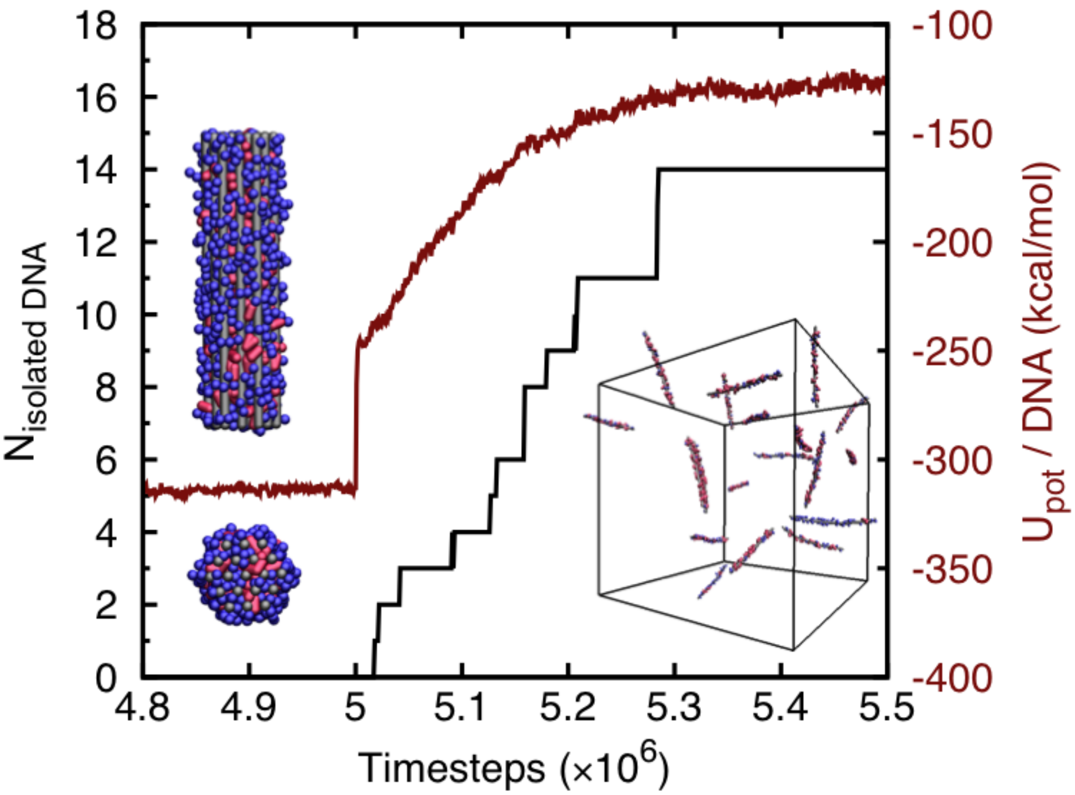

Figure S4. A pre-formed bundle (at $R_{+/-}$ = 0.5) made of 19 DNA fragments kept fixed and completely rigid for 5x10^6 timesteps on an hexagonal lattice a_H ~ 14 Å to allow complexation with protamines and counterions (left inset) undergoes a complete dissolution over time through molecular dynamics. The final state (right inset, after 15 x 10^6 timesteps) corresponds to mainly isolated DNA decorated with protamines. The increasing number of isolated DNA fragments (black) and the corresponding potential energy per DNA (red) during the first stages of the process are shown.

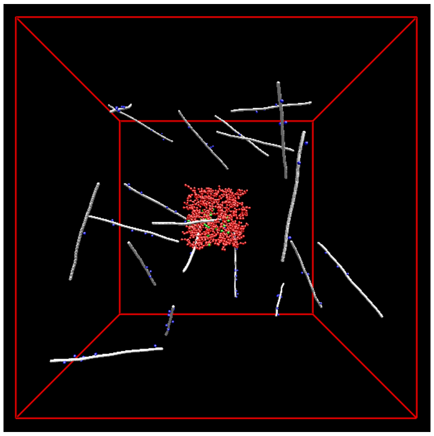

Movie S1. Inhomogeneous mixing conditions consisting of a small cubic droplet of volume v = V/100 initially injected into a DNA solution of volume V, at $R_{+/-}$ = 0.5 (N_{DNA} = 20, Figure 2B). First stage of the time evolution showing the bundle formation (only DNA, protamines and counterions adsorbed onto DNA are displayed).

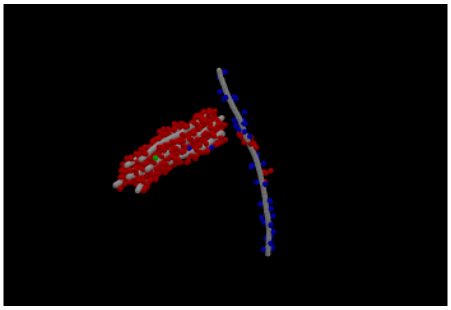

Movie S2. Transient aggregation event between a negatively charged bundle and an isolated poorly decorated (by protamines) DNA resulting in a protamine transfer and in an increase of the charge of the bundle. Inhomogeneous mixing conditions at $R_{+/-}$ = 0.5 (Figures 2B and 2C).

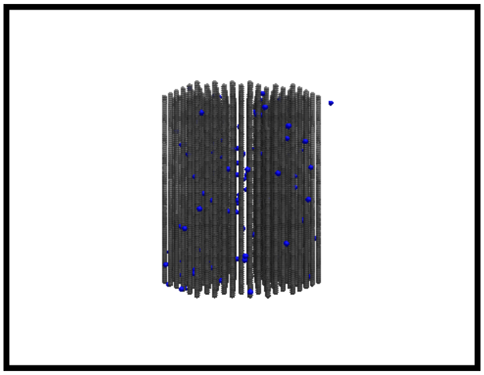

Movie S3. Longitudinal fluctuations of DNA fragments in a pre-formed bundle stabilized at $R_{+/-} = 2$ ($N_{DNA} = 91$). Only DNA and their counterions are displayed for clarity.
